# Hindering triple negative breast cancer progression by targeting endogenous interleukin‐30 requires IFNγ signaling

**DOI:** 10.1002/ctm2.278

**Published:** 2021-01-24

**Authors:** Carlo Sorrentino, Stefania Livia Ciummo, Luigi D'Antonio, Paola Lanuti, Scott I. Abrams, Zhinan Yin, Li‐Fan Lu, Emma Di Carlo

**Affiliations:** ^1^ Department of Medicine and Sciences of Aging “G. d'Annunzio” University Chieti Italy; ^2^ Anatomic Pathology and Immuno‐Oncology Unit, Center for Advanced Studies and Technology (CAST) “G. d'Annunzio” University Chieti Italy; ^3^ Department of Immunology Roswell Park Cancer Institute (RPCI) Buffalo New York USA; ^4^ The First Affiliated Hospital, Biomedical Translational Research Institute, Guangdong Province Key Laboratory of Molecular Immunology and Antibody Engineering Jinan University Guangzhou China; ^5^ Division of Biological Sciences, Center for Microbiome Innovation and Moores Cancer Center University of California San Diego California USA

**Keywords:** IFNγ, interleukin‐30, triple negative breast cancer, tumor microenvironment

## Abstract

- IL30mRNA expression is associated with the TNBC subtype.
- IL30 boosts proliferation and migration of TNBC cells and reshapes their immunity gene expression profile.
- The lack of endogenous IL30 hinders TNBC growth and progression and prolongs host survival.
- TNBC growth inhibition, due to the lack of endogenous IL30, requires INFγ production by T and NK cells.

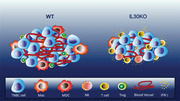

Dear Editor,

We recently identified interleukin (IL) 30 as a chief regulator of prostate and breast cancer (BC) microenvironments.^1^, ^2^, ^3^ Produced by cancer and infiltrating myeloid‐derived cells (MDC), IL30 primes transcriptional activation of oncogenes and metastasis‐related genes and promotes cancer stem‐like cell proliferation and migration, driving tumor progression.

Identified as a partner of the Epstein‐Barr virus‐induced gene 3 (EBI3) to form the heterodimeric cytokine IL27,^4^ IL30 can behave as a self‐standing cytokine, which signals via IL6Rα (CD126) by recruiting a gp130 (CD130) homodimer.^5^

IL30 expression by tumor‐ and draining lymph node (LN)‐infiltrating leukocytes, is frequent in triple‐negative (TN) BC, one of the deadliest malignancies, and has been associated with recurrence and worse overall survival.^3^

Bioinformatic analyses of microarray data, obtained from 1699 BC cases included in the METABRIC cohort (Supplementary Methods), established a positive correlation between *IL30*mRNA expression and TNBC, *versus* Luminal A (*P *< .0001), *versus* Luminal B *(P *= .045) and *versus* Normal‐like *(P *= .047) BCs (Figure 1A). Expression of *IL30*mRNA in HER2^+^BC was comparable to that of TNBC and significantly (*P *< .001) higher than in Luminal A, whereas no difference was found between the remaining BC subtypes. Although the prevalence of TNBC (15‐20% of all BCs) may seem low, the high incidence of BC, estimated at 2 000 000 cases per year worldwide,^6^ means that ∼30 000 patients (7.54% of TNBCs) will be newly diagnosed with IL30^+^ TNBCs each year. Since IL30 expression also involves 5% of HER2^+^ (10 000 cases), 3.47% of Luminal B (12 000 cases), 2.14% of Normal‐like (2 000 cases), and 0.74% of Luminal A (6 000 cases) BCs, the total number of patients with IL30^+^ BCs will be ∼60,000.

**FIGURE 1:**
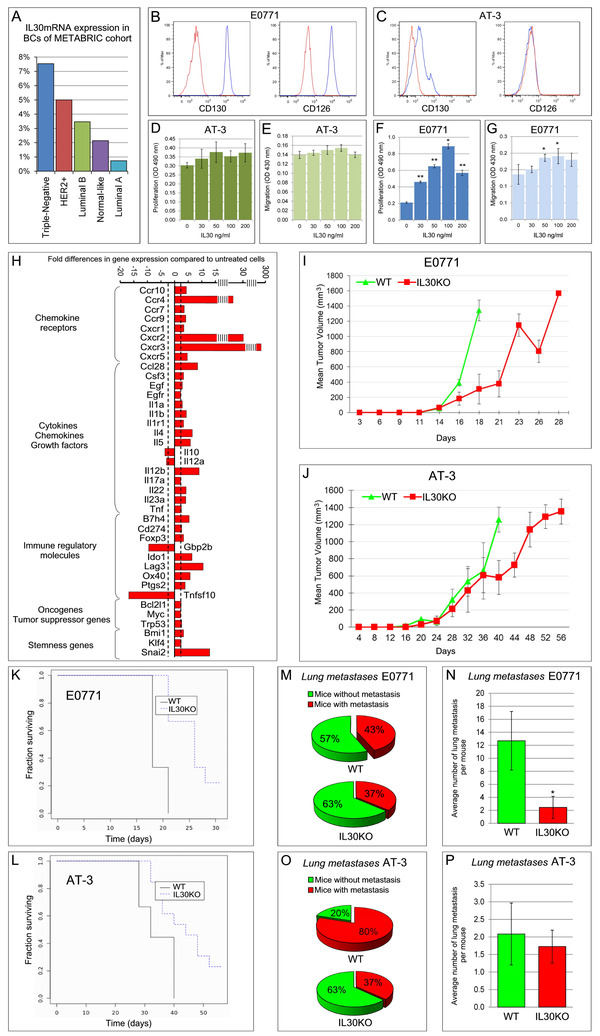
IL30 expression in human TNBC and effects of IL30 on murine TNBC cell lines in vitro and in vivo. A, *IL30*mRNA expression in human BC subtypes from the METABRIC cohort. The histogram represents the percentage of *IL30* over‐expressing cases (Z‐score > 2) out of the total number of cases for each BC subtype. B and C, Cytofluorimetric analyses of gp130 (CD130) and IL6Rα (CD126) expression in E0771 (B) and in AT‐3 cells (C). Blue profiles: specific markers; red profiles: isotype controls. Experiments were performed in triplicate. D, MTT assay of AT‐3 cells, 48 hours after rmIL30 treatment. Data are representative of three independent experiments. Results are expressed as mean ± SD. ANOVA: *P *= .315. E, Number of migrating and invading AT‐3 cells, 48 hours after rmIL30 treatment. Results are expressed as mean ± SD. ANOVA: *P *= .118. F, MTT assay of E0771 cells, 48 hours after rmIL30 treatment. Data are representative of three independent experiments. Results are expressed as mean ± SD. ANOVA: *P *< .001. *P < .05, Tukey HSD Test compared with 0, 30, 50, and 200 ng/mL. **P < .05, Tukey HSD Test compared with 0 ng/mL. G, Number of migrating and invading E0771 cells, 48 hours after rmIL30 treatment. Results are expressed as mean ± SD. ANOVA: *P *= .011. *P < .05, Tukey HSD Test compared with 0 ng/mL. H, Cancer inflammation and immunity crosstalk PCR Array reveals the fold differences of individual mRNAs between E0771 cells treated and untreated with rmIL30 (red bars). Pooled results ± SD are from two experiments performed in duplicate. A significant threshold of a two‐fold change in gene expression corresponded to a *P *< .001. I and J, Mean volume of tumors developed after orthotopic implantation of E0771 (I) and AT‐3 cells (J) in WT or in *IL30KO* mice. Student's *t*‐test: *P *< .01 *versus* WT mice. Results from *B6 EIIa‐cre* mice and *p28^f/f^* mice were comparable to those obtained in WT mice (Fisher Exact Probability Test: *P *> .99). K and L, Kaplan‐Meier survival curves of E0771 (K) and AT‐3 (L) tumor bearing WT and *IL30KO* mice. Log‐rank test: *P *= .0014 (K) and .012 (L). M, Percentage of WT and *IL30KO* mice, bearing E0771 tumors, with (red) and without (green) lung metastasis. Fisher Exact Probability Test: *P *= .792. N, Average number of lung metastasis per mouse developed in WT and *IL30KO* mice bearing E0771 tumors. *P = .004, Student's *t*‐test compared with WT mice. The number of metastases per mouse did not correlate with the tumor size, as determined by Pearson correlation coefficient (*r *= .002). O, Percentage of WT and *IL30KO* mice, bearing AT‐3 tumors, with (red) and without (green) lung metastasis. Fisher Exact Probability Test: *P *= .0014. The number of mice which developed metastasis did not correlate with the tumor size, as determined by Spearman's rank correlation coefficient (*ρ *= .203). P, Average number of lung metastasis per mouse developed in WT and *IL30KO* mice bearing AT‐3 tumors. Student's *t*‐test: *P *= .129

To find out whether IL30 targeting in the host environment might affect TNBC behavior, murine TNBC cell lines, E0771, and AT‐3, were first tested for their responsiveness to IL30 and then implanted into syngeneic, *EIIa‐p28^f/f^*, IL30 conditional knockout^7^ (*IL30KO)* hosts (Supplementary Methods).

Only E0771 cells expressed both IL30 receptor (R) chains (Figures 1B and 1C; Figure S1 and Supplementary Methods). Both cell lines did not release IL30, which excludes autocrine effects. AT‐3 cells proved unresponsive to rmIL30 in proliferation and motility tests (Figures 1D and 1E), and immunity gene expression profiling. By contrast, rmIL30 increased proliferation (ANOVA: *P *< .001) and migration (ANOVA: *P *= .0108) of E0771 cells (Figures 1F and 1G), and reshaped their immunity gene expression (Supplementary Methods; Figure 1H) up‐regulating inflammatory mediators, such as *Il1β*, *Il4*, *Il5*, *Il17a*, *Csf3*, *Egf*, *Il23*, *Il22*, *Ccl28* and chemokine receptors, especially, *Ccr4, Cxcr5, Cxcr2*, and *Cxcr3*, which promote invasive migration of cancer cells.^8^ IL30 also upregulated *Ptgs2*, *Ido1*, *Cd274/Pd‐l1*, *B7h4*, and *Lag3*,^9^ which mediate tumor immune escape, along with *Bcl2l1*, *Myc*, *Trp53*, *Klf4*, *Bmi1*, and, especially, *Snai2*, which fosters tumor progression.^10^

The tumor‐promoting effects on TNBC cells endowed with the IL30R, led us to speculate that targeting IL30 might inhibit IL30‐responsive tumors. However, when orthotopically implanted into *IL30KO* mice, both IL30‐responsive and unresponsive TNBC cell lines gave rise to tumors that grew slower (Student's *t*‐test, *P *< .01) than in WT mice, and reached, 18 and 40 days later, respectively, a mean tumor volume (MTV) that was lower than that of WT mice (E0771 tumors: 309 ± 194 mm^3^ in *IL30KO* *versus* 1342 ± 137 mm^3^ in WT; AT‐3 tumors: 580 ± 199 mm^3^ in *IL30KO* *versus* 1257 ± 145 mm^3^ in WT; Student's *t*‐test: *P *= .00005 and *P *= .0020, respectively; Figures 1I and 1J). In both experiments, the survival of tumor‐bearing *IL30KO* mice was considerably longer (log rank test: *P *< .05; Figures 1K and 1L).

Among E0771 tumor‐bearing mice, the number of *IL30KO* mice, which developed lung metastasis (11/30; 37%) was comparable to that of WT mice (13/30, 43%) (Figure 1M), however the average number of metastasis per mouse was lower in *IL30KO* mice (3 *versus* 13) (Student's *t*‐test: *P *= .004; Figure 1N).

Among AT‐3 tumor‐bearing mice, only 11 out of 30 (37%) of *IL30KO* mice developed lung metastasis *versus* 24 out of 30 (80%) of WT mice (Fisher Exact Test: *P *= .0014; Figure 1O), whereas no difference in the average number of metastases per mouse was found between the two groups (Figure 1P).

In WT mice, both E0771 and AT‐3 tumors were well vascularized and infiltrated by IL30 expressing leukocytes, mostly MDCs and macrophages, that were lacking in tumors developed in *IL30KO* mice (Supplementary Methods and Tables S1 and S2; Figures 2A‐2E). The absence of EBI3 excluded IL27 production in the microenvironment (Figure S2). In *IL30KO* mice, both types of tumors were poorly vascularized with necrotic‐hemorrhagic features, whereas only E0771 tumors showed a lower proliferation index (Figures 2A‐2C; Table S2). In *IL30KO* mice, both tumor models revealed a scanty CD11b^+^Gr‐1^+^MDC and F4/80^+^macrophage infiltrate, and a reduced CD4^+^Foxp3^+^T regulatory cell content, whereas CD4^+^T, CD8^+^T, and NKp46^+^ cells were increased when compared with controls (Figures 2D and 2E).

**FIGURE 2:**
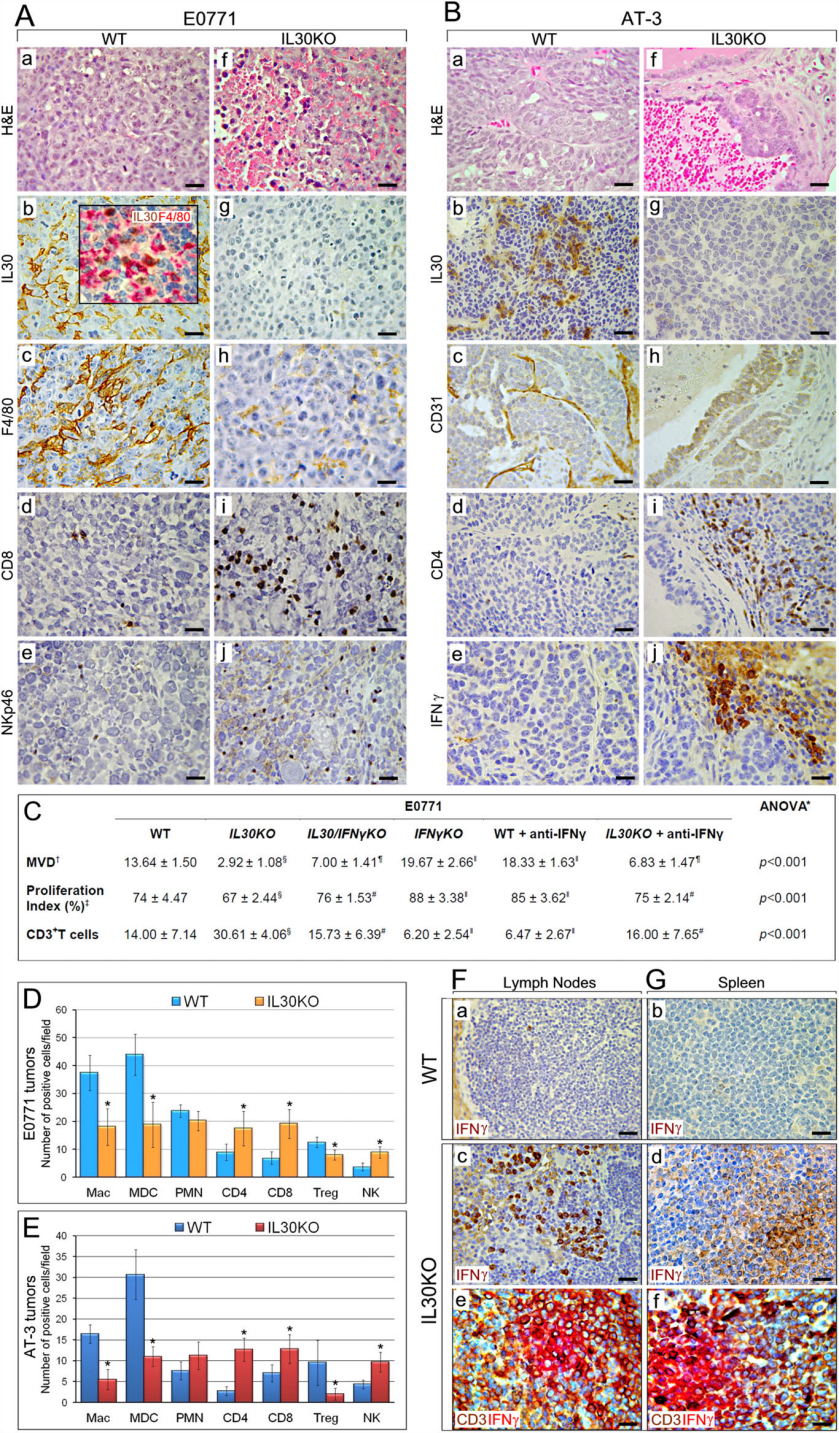
Immuno‐pathological features of TNBCs developed in *IL30KO* mice. A and B, Hematoxylin and Eosin (H&E) stained sections and immunohistochemical characterization of E0771 tumors (A) and AT‐3 tumors (B) developed in WT (a, b, c, d, e) and in *IL30KO* (f, g, h, i, j) mice. Magnification: X400. Scale bars: 30 µm. C, Microvessel density (MVD), proliferation, and T lymphocyte infiltration of E0771 tumors. *One‐way ANOVA for comparisons between the six mouse groups. ^†^MVD: mean ± SD of CD31 positive vessels/field (85431.59 µm^2^). ^‡^Proliferation Index (%): mean percentage ± SD of PCNA positive cells/number of total cells per field (85431.59 µm^2^). ^§^ *P *< .01, Tukey HSD Test compared with tumors in WT, *IL30/IFNγKO*, *IFNγKO*, WT + anti‐IFNγ Abs and *IL30KO* + anti‐IFNγ Abs. *^¶^P *< .01, Tukey HSD Test compared with tumors in WT, *IL30KO, IFNγKO*, and WT + anti‐IFNγ Abs. ^#^ *P *< .01, Tukey HSD Test compared with tumors in *IL30KO*, *IFNγKO*, and WT + anti‐IFNγ Abs. ^‖^ *P *< .01, Tukey HSD Test compared with tumors in WT, *IL30KO*, *IL30/IFNγKO*, and *IL30KO* + anti‐IFNγ Abs. D and E, Immune cell counts in E0771 (D) and AT‐3 (E) tumors developed in WT and in *IL30KO* mice. Results are expressed as mean ± SD of positive cells/field evaluated at X400 (85431.59 µm^2^ field) by immunohistochemistry. *P < *.01*, Student's *t*‐test compared with tumors in WT mice. F and G, Immunohistochemical detection of IFNγ (brown) in tumor draining lymph nodes (a, c, e) and in the spleen (b, d, f) of E0771 tumor bearing WT (a, b) and *IL30KO* (c, d, e, f) mice. The double staining reveals the frequent co‐localization (brick color) of IFNγ (red) with CD3^+^T cells (brown), both in the lymph nodes (e) and spleen (f) of E0771 tumor bearing *IL30KO* mice. Magnification: X400. Scale bars: 30 µm

IFNγ expression was found in tumors, LNs and spleens of tumor‐bearing *IL30KO* mice and largely co‐localized with CD3^+^T lymphocytes (Figures 2B, 2F and 2G; Figures S3A and S3B, and Table S3), although the contribution of myeloid cells cannot be completely excluded. Specifically, CD3^+^CD8^+^, CD3^+^CD4^+^, and CD3^−^NKp46^+^ cells, to a lesser extent, were the source of IFNγ production in the tumor microenvironment (Figures 3A and 3B).

**FIGURE 3:**
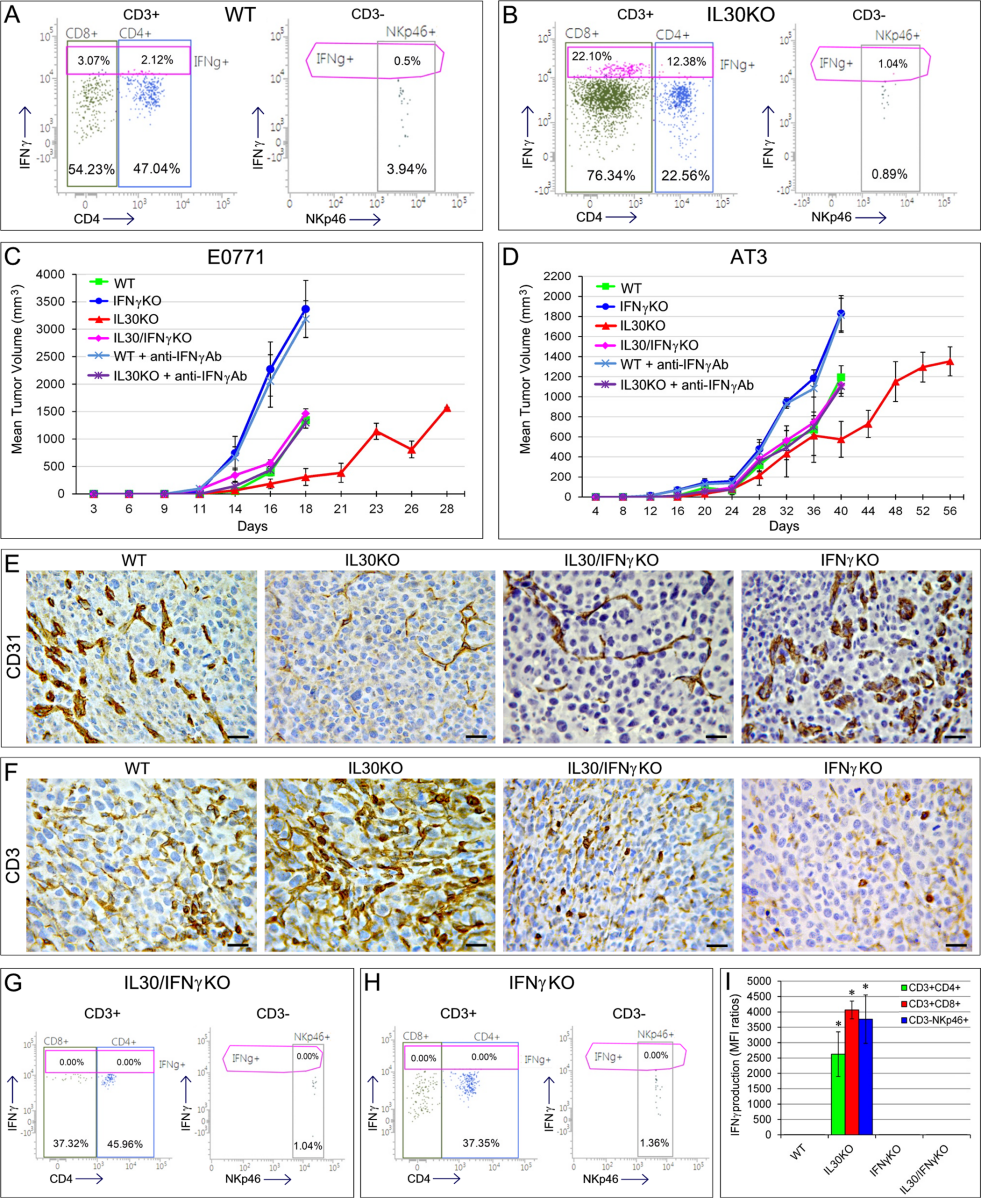
Growth curves and immuno‐pathological features of TNBCs developed in *IL30KO*, *IFNγKO*, *IL30/IFNγKO* mice, and in WT or *IL30KO* mice treated with neutralizing anti‐IFNγ Abs. A and B, Flow cytometry analysis of IFNγ production, by tumor infiltrating cells, in two representative tumors developed in WT (A) and *IL30KO* (B) mice injected with E0771 cells. After exclusion of dead cells (7‐AAD+), CD3^+^CD4^+^ (blue), CD3^+^CD8^+^ (green), and CD3^−^NKp46^+^ (grey) nucleated cells were gated and analyzed for IFNγ expression (purple). The percentages of CD4^+^cells and CD8^+^cells of parent CD3^+^population, percentage of NKp46^+^cells of CD3^−^parent population and percentage of IFNγ^+^cells are reported. Isotype controls were used to assess the background, and experiments were performed in triplicate. C and D, Mean volume of tumors (MTV) developed after orthotopic implantation of E0771 (C) and AT‐3 (D) cell lines in *IL30/IFNγ*KO mice, *IFNγ*KO mice, *IL30*KO mice, WT mice, and WT or *IL30*KO mice treated with anti‐IFNγ Abs. ANOVA: *P <* *.0001*. Tukey's HSD test: *P <* *.01* (*IFNγKO* mice and WT mice treated with anti‐IFNγ Abs *versus* *IL30*KO, *IL30/IFNγKO*, *IL30KO* mice treated with anti‐IFNγ Abs and WT mice; *IL30*KO mice *versus* *IFNγKO* mice, WT mice treated with anti‐IFNγ, *IL30/IFNγKO* mice, *IL30*KO mice treated with anti‐IFNγ Abs and WT mice). The growth curves of both E0771 and AT‐3 tumors, developed in *IL30KO* mice treated with anti‐IFNγ Abs, were comparable to those observed in *IL30/IFNγKO* mice (MTV of E0771 tumors in *IL30KO* mice treated with anti‐IFNγ Abs: 1298.59 ± 103.54 mm^3^; MTV of E0771 tumors in *IL30/IFNγKO* mice: 1464.16 ± 87.34 mm^3^; Tukey HSD Test: *P *= .18. MTV of AT‐3 tumors in *IL30KO* mice treated with anti‐IFNγ Abs: 1103.23 ± 98.46 mm^3^; MTV of AT‐3 tumors in *IL30/IFNγKO* mice: 1118.30 ± 85.98 mm^3^; Tukey HSD Test: *P *= .90). The growth of tumors in WT mice treated with anti‐IFNγ Abs was comparable to that observed in *IFNγKO* mice (MTV of E0771 tumors in WT mice treated with anti‐IFNγ Abs: 3186.89 ± 333.71 mm^3^; MTV of E0771 tumors in *IFNγKO* mice: 3369.92 ± 520.94 mm^3^; Tukey HSD Test: *P *= .10. MTV of AT‐3 tumors in WT mice treated with anti‐IFNγ Abs: 1811.37 ± 169.94 mm^3^; MTV of AT‐3 tumors in *IFNγKO* mice: 1831.85 ± 176.00 mm^3^; Tukey HSD Test: *P *= .90). E, The vascular network of E0771 tumors developed in WT (a), *IL30KO* (b), *IL30/IFNγKO* (c), and *IFNγKO* (d) mice, as assessed by CD31 immunostaining. Magnification: X400. Scale bars: 30 µm. F, T lymphocyte infiltrate in E0771 tumors developed in WT (a), *IL30KO* (b)*, IL30/IFNγKO* (c), and *IFNγKO* (d) mice, as assessed by CD3 immunostaining. Magnification: X400. Scale bars: 30 µm. G and H, Flow cytometry analysis of IFNγ production, by tumor infiltrating cells, in two representative tumors developed in *IFNγKO* and *IL30/IFNγKO* mice, shows the absence of IFNγ in tumor infiltrating CD3^+^CD4^+^, CD3^+^CD8^+^, and CD3^−^NKp46^+^ cells. The percentages of CD4^+^cells and CD8^+^cells of parent CD3^+^population, percentage of NKp46^+^cells of CD3^−^parent population, and percentage of IFNγ^+^cells are reported. Isotype controls were used to assess the background and experiments were performed in triplicate. I, The production of IFNγ, measured as mean fluorescence intensity (MFI) ratios, detected in CD3^+^CD4^+^, CD3^+^CD8^+^, and CD3^−^NKp46^+^ cells infiltrating the tumors developed in WT, *IL30KO, IFNγKO, and IL30/IFNγKO* mice. MFI ratios were calculated by dividing the MFI of IFNγ^+^cell population by the MFI of the negative/isotype control. Five tumor samples per experimental group were examined

To investigate the role of IFNγ in the anti‐tumor effects due to the lack of IL30, we generated *IL30/IFNγKO* mice (Supplementary Methods and Figure S4A, S4B, and S4C) and selectively blocked, in IL30KO mice, IFNγ pathway with neutralizing anti‐IFNγ Abs.

The growth of both TNBCs was restored in *IL30/IFNγKO* or in *IL30KO* hosts in which the IFNγ pathway was blocked.

In *IL30/IFNγKO* hosts, E0771 and AT‐3 cells gave rise to tumors that reached a MTV of 1470 ± 88 and 1073 ± 135 mm^3^, respectively, that was significantly higher (ANOVA: *P *< .001; Tukey HSD Test: *P *< .01) than that of tumors developed in *IL30KO* mice and comparable to that of control tumors in WT mice (Figures 3C and 3D). The proliferation index of tumors developed in *IL30/IFNγKO* mice was comparable to control tumors. In both E0771 and AT‐3 tumors, which developed in *IL30/IFNγKO* mice, the microvessel density was higher (ANOVA: *P <* .001; Tukey HSD Test: *P <* .01) than in tumors developed in *IL30KO* mice, and comparable to control tumors. By contrast, CD3^+^T lymphocytes were drastically reduced when compared with tumors developed in *IL30KO* (ANOVA: *P *< .001; Tukey HSD Test: *P *< .01) and comparable to control tumors (Table S2; Figures 2C, 3E, and 3F). Thereby, abrogation of IFNγ signaling enhances tumor vascularization and prevents the intra‐tumoral influx of T lymphocytes.^11^

IFNγ, evaluated by flow cytometry (Supplementary Methods), was absent in tumors developed in *IL30/IFNγKO and IFNγKO* mice (Figures 3G and 3H), whereas it was produced by CD3^+^CD4^+^, CD3^+^CD8^+^, and CD3^−^NKp46^+^ cells infiltrating tumors developed in *IL30KO mice* (Figure 3I).

The growth, vascularization, and T lymphocyte content of both E0771 and AT‐3 tumors, developed in *IL30KO* mice treated with anti‐IFNγ Abs, were comparable to those observed in *IL30/IFNγKO* mice (MTV of E0771 tumors: Tukey HSD Test: *P *= .18; MTV of AT‐3 tumors: Tukey HSD Test: *P *= .90; Figures 2C, 3C, and 3D; Table S2).

Overall, the lack of endogenous IL30 triggers IFNγ production by T and NK cells, hinders TNBC progression and leads to improved survival. This study consolidates our recent findings^12^ and provides the proof of concept that IL30 is a valuable target to improve immunotherapy and life expectancy in TNBC patients.

## CONFLICT OF INTEREST

The authors declare that there is no conflict of interest that could be perceived as prejudicing the impartiality of the research reported.

## ETHICS APPROVAL AND CONSENT TO PARTICIPATE

Animal procedures were performed in accordance with the European Community guidelines and approved by the Institutional Animal Care Committee of "G. d'Annunzio" University and the Italian Ministry of Health (authorization number: 892/2018‐PR).

## AUTHOR CONTRIBUTIONS

Emma Di Carlo was responsible for the study design and supervision and wrote the paper; Carlo Sorrentino, Stefania Livia Ciummo, Luigi D'Antonio, Paola Lanuti performed the experiments; Emma Di Carlo, Carlo Sorrentino, Stefania Livia Ciummo performed data analysis; Li‐Fan Lu and Zhinan Yin provided *IL30KO* mice; Scott I. Abrams provided the AT‐3 cell line; and all authors approved the final version of the manuscript.

## CONSENT FOR PUBLICATION

All authors have read the manuscript and approved its submission to *Clinical and Translational Medicine*.

## DATA AVAILABILITY STATEMENT

The data that support the findings of this study are available from Emma Di Carlo upon reasonable request.

## Supporting information

Supplementary Figures

Supplementary Tables

Supplementary Information
