# Supplementary material for: Hindering triple negative breast cancer progression by targeting endogenous interleukin‐30 requires IFNγ signaling
Source: Clin Transl Med. 2021 Jan 24;11(2):e278. doi: 10.1002/ctm2.278 (PMC7828256; doi:10.1002/ctm2.278)
Supplement: Supplementary file 1 — Supplementary Figures [file CTM2-11-e278-s001.pdf]

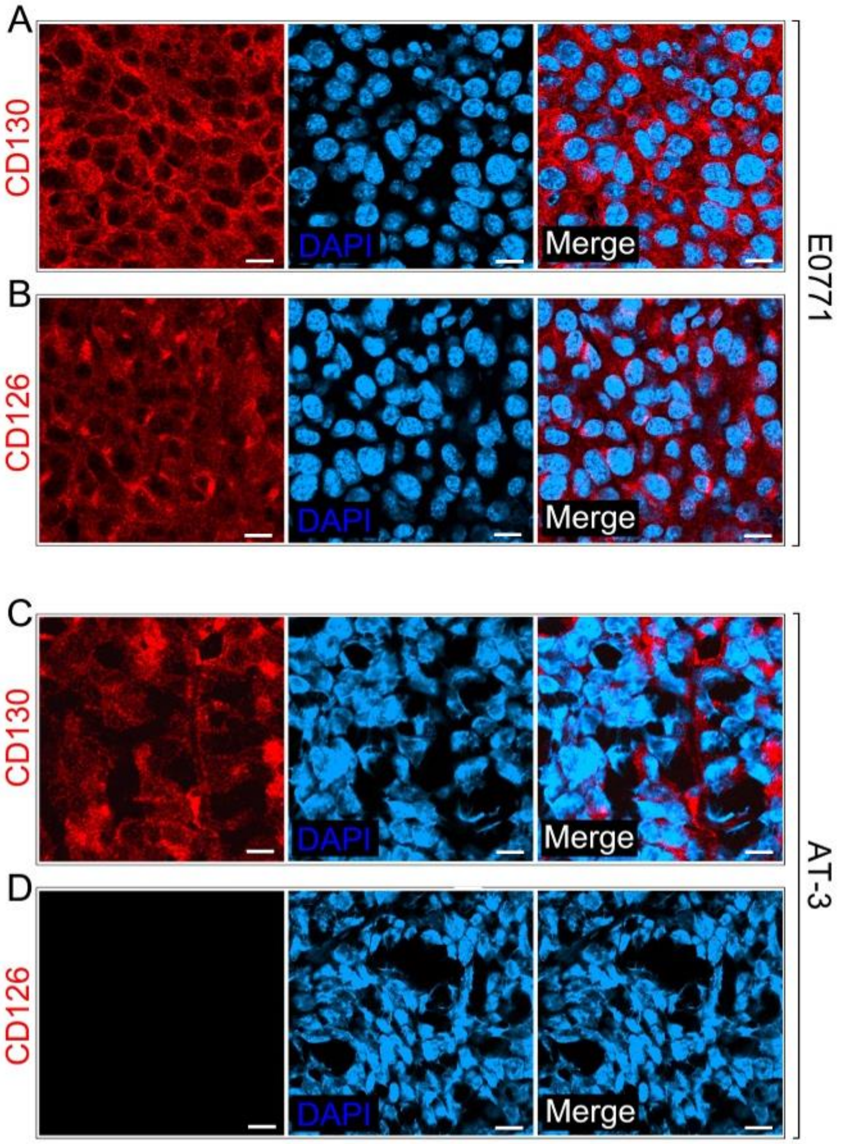

SUPPLEMENTARY FIGURE S1 Laser scanning confocal analyses of gp130 (CD130) and IL6Rα (CD126) in E0771 and AT-3 tumors growing in WT mice.

A and **B**. Tumors developed after orthotopic implantation of E0771 cells in C57BL/6J mice show cancer cell expression (red stained) of both CD130 (**A**) and CD126 (**B**).

C and **D**. Tumors developed after orthotopic implantation of AT-3 cells in C57BL/6J mice show cancer cell expression (red stained) of CD130 (**C**), whereas CD126 was absent (**D**). DAPI stained nuclei in blue. Magnification: A, B and C, X630; D, X400. Scale bars: A, B and C, 10 µm; D, 30 µm.

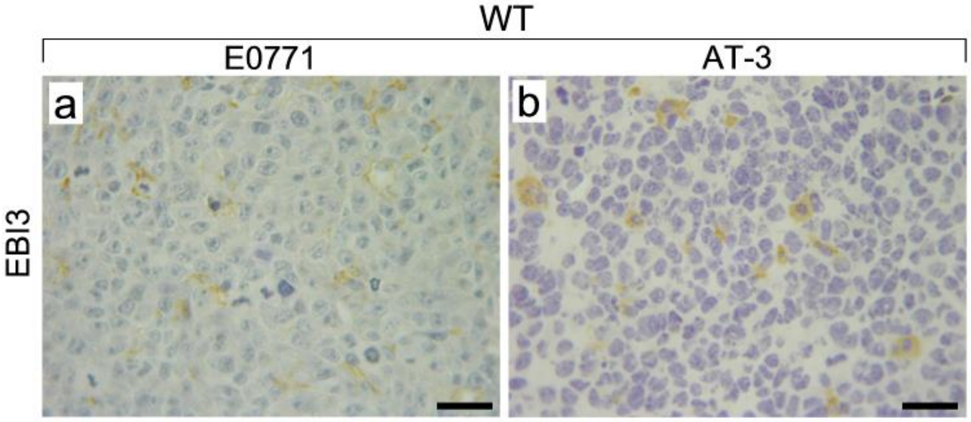

SUPPLEMENTARY FIGURE S2 EBI3 immunostaining in E0771 **(a)** and AT-3 **(b)** tumors developed in WT mice. Magnification: X400. Scale bars: 30 µm.

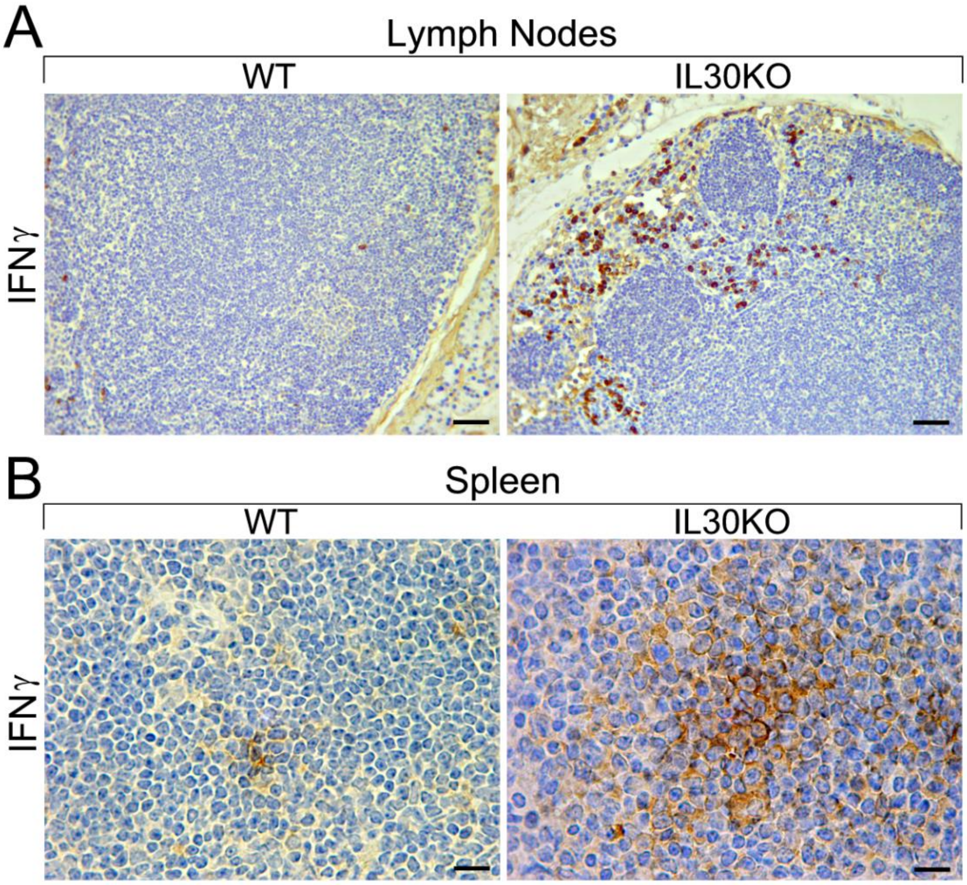

SUPPLEMENTARY FIGURE S3 Interferon gamma immunostaining in tumor draining lymph nodes **(A)** and spleen **(B)** of WT and *IL30KO* mice bearing AT-3 tumors. Magnification: A, X200; B, X400. Scale bars: A, 50 µm; B, 30 µm.

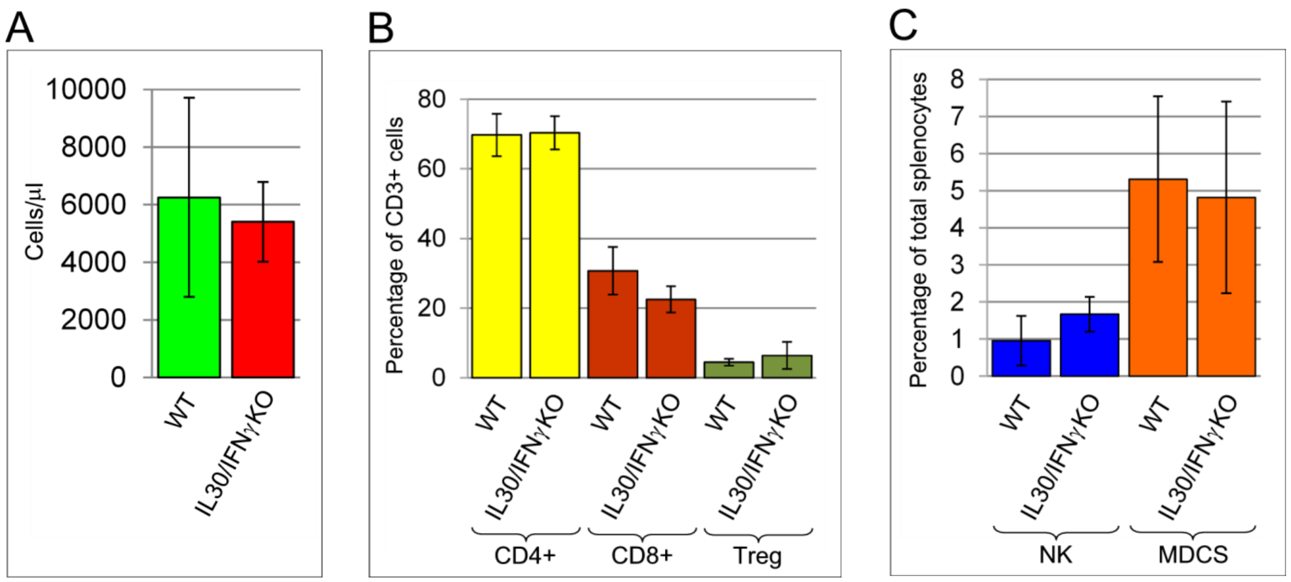

SUPPLEMENTARY FIGURE S4 Characterization of *IL30/IFNγKO* mice by flow cytometry analyses of the composition of the main splenic immune cell populations

A. Total number of cells in the spleens of WT and *IL30/IFNγKO* mice (five mice per group were analysed). Results are expressed as mean ± SD of viable cells evaluated by flow cytometry, using 7-amino-actinomycin D (7-AAD) staining. Student's *t*-test: *p*=0.723.

B. Flow cytometry analysis of $CD3^+CD4^+$, $CD3^+CD8^+$, and $CD3^+CD4^+CD25^+Foxp3^+$ (T regulatory) cell populations in the spleens of WT and *IL30/IFNγKO* mice (five mice per group were analyzed). Results are expressed as mean ± SD. Student's *t*-test: *p*=0.906 ($CD3^+CD4^+$), *p*=0.162 ($CD3^+CD8^+$), *p*=0.396 (Tregs).

C. Flow cytometry analysis of $NKp46^+$ (NK cells) and $CD11b^+Gr\text{-}1^+$ (Myeloid Derived Suppressor Cells) populations in the spleens of WT and *IL30/IFNγKO* mice (five mice per group were analyzed). Results are expressed as mean ± SD. Student's *t*-test: *p*=0.214 (NK), *p*=0.816 (MDSCs).

Therefore, no significant differences were observed in the composition of $CD3^+CD4^+$, $CD3^+CD8^+$, Treg ($CD3^+CD4^+CD25^+Foxp3^+$), NK ($NKp46^+$) and myeloid derived suppressor ($CD11b^+Gr\text{-}1^+$) cells in the spleen between WT and *IL30/IFNγKO* mice.
