# Supplementary material for: Hindering triple negative breast cancer progression by targeting endogenous interleukin‐30 requires IFNγ signaling
Source: Clin Transl Med. 2021 Jan 24;11(2):e278. doi: 10.1002/ctm2.278 (PMC7828256; doi:10.1002/ctm2.278)
Supplement: Supplementary file 2 — Supplementary Tables [file CTM2-11-e278-s002.pdf]

SUPPLEMENTARY TABLE S1 Antibodies used in immunostaining

Antibody	Clone	Origin	Research Resource Identifiers (RRIDs)	Source
CD3		Rabbit	RRID:AB_2335677	Agilent, Santa Clara, CA, USA
CD4	YTS191.1.2	Rat	RRID:AB_323559	Bio-Rad, Hercules, CA, USA
CD8	YTS169.4	Rat	RRID:AB_322770	Bio-Rad, Hercules, CA, USA
CD11b	EPR1344	Rabbit	RRID:AB_2650514	Abcam, Cambridge, UK
CD31	SZ31	Rat	RRID:AB_2631039	Dianova, Hamburg, Germany
EBI3	DNT27	Rat	RRID:AB_10903507	Abcam, Cambridge, UK
F4/80	Cl:A3-1	Rat	RRID:AB_323279	Bio-Rad, Hercules, CA, USA
Foxp3	FJK-16s	Rat	RRID:AB_467575	Thermo Fisher, Waltham, MA, USA
Gr-1	RB6-8C5	Rat	RRID:AB_394638	BD Biosc., Franklin Lakes, NJ, USA
IFNγ	LLO6Z	Mouse	RRID:AB_1123595	Santa Cruz, Dallas,TX, USA
IL30(IL27p28)		Goat	RRID:AB_355012	R&D Systems, Minneapolis, MN, USA
Ly-6G	1A8	Rat	RRID:AB_1089179	BioLegend, San Diego, CA, USA
NKp46		Rabbit	RRID:AB_10767953	Biorbyt, Cambridge, UK
PCNA	PC10	Mouse	RRID:AB_2160651	Agilent, Santa Clara, CA, USA

SUPPLEMENTARY TABLE S2

	AT-3						ANOVA*
	**WT**	***IL30KO***	***IL30/IFNγKO***	***IFNγKO***	**WT + anti-IFNγ**	***IL30KO* + anti-IFNγ**	
**MVD†**	14.00 ± 3.00	6.67 ± 1.87§	10.57 ± 0.85¶	22.50 ± 2.24‖	21.50 ± 2.24‖	9.93 ± 1.14¶	p<0.001
**Proliferation Index (%)‡**	68 ± 2.74	64 ± 3.65	71 ± 1.73#	82 ± 2.23‖	81 ± 1.82‖	70 ± 4.89#	p<0.001
**$CD3^+$T cells**	11.75 ± 4.59	27.57 ± 6.97§	10.07 ± 3.22#	4.71 ± 2.55‖	4.29 ± 1.59‖	10.00 ± 4.28#	p<0.001

*One-way ANOVA for comparisons between the six mouse groups.
†MVD: microvessel density expressed as mean ± SD of CD31 positive vessels/field (85431.59 μm^2).
‡Proliferation Index (%): mean percentage ± SD of PCNA positive cells/number of total cells per field (85431.59 μm^2).
§*p*<0.01, Tukey HSD Test compared with tumors in WT, *IL30/IFNγKO*, *IFNγKO*, WT + anti-IFNγ and *IL30KO* + anti-IFNγ Abs.
¶*p*<0.01, Tukey HSD Test compared with tumors in WT, *IL30KO*, *IFNγKO* and WT + anti-IFNγ Abs.
#*p*<0.01, Tukey HSD Test compared with tumors in *IL30KO*, *IFNγKO* and WT + anti-IFNγ Abs.
‖*p*<0.01, Tukey HSD Test compared with tumors in WT, *IL30KO*, *IL30/IFNγKO* and *IL30KO* + anti-IFNγ Abs.

SUPPLEMENTARY TABLE S3

Tumors	IFNγ expression		
	**WT**	**IL30KO**	**Student's t-test†**
**E0771**			
*Tumors**	1.90 ± 1.20	18.90 ± 5.00	$p<0.001$
*Lymph Nodes**	2.90 ± 1.66	25.30 ± 4.37	$p<0.001$
*Spleen**	2.70 ± 1.34	14.10 ± 4.01	$p<0.001$
**AT-3**			
*Tumors**	1.44 ± 1.33	16.56 ± 5.48	$p<0.001$
*Lymph Nodes**	1.80 ± 1.75	18.90 ± 6.12	$p<0.001$
*Spleen**	3.50 ± 1.78	17.70 ± 7.15	$p<0.001$

*IFNγ expression values are represented as the mean percentage of positively stained areas/total area of the examined field (85431.59μm^2) at ×400.
†Student's *t*-test for comparisons between WT and *IL30KO* mice.
