# Supplementary material for: Hindering triple negative breast cancer progression by targeting endogenous interleukin‐30 requires IFNγ signaling
Source: Clin Transl Med. 2021 Jan 24;11(2):e278. doi: 10.1002/ctm2.278 (PMC7828256; doi:10.1002/ctm2.278)
Supplement: Supplementary file 3 — Supplementary Information [file CTM2-11-e278-s003.pdf]

SUPPLEMENTARY METHODS

Bioinformatic analyses

For bioinformatic analyses (cBioPortal, RRID:SCR_014555), Microarray data of The Molecular Taxonomy of BC International Consortium (METABRIC) dataset, which includes 1699 BC cases, were downloaded from the cBioportal for Cancer Genomics database (*https://www.cbioportal.org*). For each sample, the Z-score of *IL30*mRNA levels was calculated, compared to the mean of all samples in the study, and all samples with a Z-score ≥ 2 were considered *IL30*-expressing. Subsequently, the association between *IL30*mRNA expression and BC subtypes was assessed using the Fisher's exact test.

Cell cultures, ELISA, MTT and Migration assays

AT-3 cells (RRID:CVCL_VR89), derived from a mammary adenocarcinoma developed in a female MMTV-PyMT transgenic mouse (IMSR Cat# JAX:022974, RRID:IMSR_JAX:022974), and classified as TNBC cells, were provided by Prof. Scott I Abrams.[1] E0771 cells (RRID:CVCL_GR23), derived from a spontaneous mammary tumor developed in a female *C57BL/6J* mouse, were purchased from CH3BioSystems (Amherst, NY, USA) and were characterized as TNBC, specifically BLBC cells.[2] Cells were passaged for fewer than 6 months after resuscitation.

Cell line authentication: AT-3 cell line (RRID:CVCL_VR89) was authenticated by staining for characteristic markers[1] whereas E0771 cell line (RRID:CVCL_GR23) was authenticated by Short-Tandem-Repeats analysis.

AT-3 cells (RRID:CVCL_VR89) were cultured using DMEM High Glucose medium (with 10% FBS), enriched with sodium pyruvate, 2-mercaptoethanol and non-essential amino acids, and E0771 cells (RRID:CVCL_GR23) were cultured in RPMI 1640 medium, supplemented with 10% FBS.

The quantitation of IL27p28/IL30 protein in the supernatant (sup) derived from E0771 and AT-3 cells was assessed using the Mouse IL-27 p28/IL-30 Quantikine ELISA Kit (#M2728, R&D Systems, Minneapolis, MN, USA; detection sensitivity: 4.27 pg/mL), according to the manufacturer's instructions.

Cell proliferation was assessed using the CellTiter 96 AQueous One Solution Cell Proliferation Assay (#G3582, Promega, Madison, WI, USA), according to manufacturer’s instructions, in the following experimental conditions: cells untreated or treated with recombinant (r) murine (m) IL30 (#7430-ML-010, R&D Systems, Minneapolis, MN, USA) (30 ng/ml, 50 ng/ml, 100 ng/ml and 200 ng/ml). Briefly, cells were seeded on a 96-well plate, at a density of 5 $\times 10^3$ cells per well in 100 µl of serum free medium, and then were incubated for 48 hours. The assay plates were read at 490 nm using a 96-well plate reader and the proliferation was measured using untreated control cells as reference. The results are given as mean ± SD of three independent experiments carried out in triplicate.

To assess the migration capacity towards IL-30, we used the QCM 8µm Chemotaxis Assay 24-well-Colorimetric kit (#ECM508, Merck, Darmstadt, Germany), according to manufacturer’s instructions. Briefly, cells were starved for 48 hours, and then seeded into a Boyden Chamber Insert at a concentration of 2.5×10^5 cells per insert. Subsequently, 300 µl of serum free medium with or without rmIL-30 at different concentrations (30 ng/ml, 50 ng/ml, 100 ng/ml and 200 ng/ml) were added in the lower chamber. Cells were incubated for 48 hours and their migration ability was assessed by spectrophotometer. Serum free medium was used as control and the results were given as mean ± SD of three independent experiments carried out in triplicate.

Mouse studies

Animal procedures were performed in accordance with the European Community guidelines, and approved by the Institutional Animal Care Committee of "G. d'Annunzio" University and the Italian Ministry of Health (Authorization n. 892/2018-PR).

C57BL/6J (WT) mice (RRID:IMSR_JAX:000664) were purchased from Envigo (Indianapolis, IN, USA) and B6.129S7-*Ifng*^{tm1Ts}/J *(IFNγKO)* (RRID:IMSR_JAX:002287) mice from Charles River Laboratories (Wilmington, MA, USA). *EIIa-p28*$^{f/f}$, i.e. *IL27p28*-null (*IL30KO*) mice,[3] were provided by Prof. Zhinan Yin (Jinan University, Guangzhou, Guangdong, China) and Prof. Li-Fan Lu (University of California, San Diego, La Jolla, CA, USA). The *IL30/IFNγKO* strain was generated in our laboratory by crossing *IFNγKO*[4] and *IL30KO* mice (both on *C57BL/6J* background) for three rounds of breeding. These mice were healthy and fertile, phenotypically grossly normal, and were born at the expected Mendelian ratio. Therefore, *IL30/IFNγKO* mice appeared normal in a naïve state.

Mouse genotyping was carried out using tail digestion, followed by genomic DNA PCR and gel electrophoresis. To demonstrate the *IL30* gene knockout, we used the following primers, designed by Prof. Zhinan Yin: wild type forward, 5'-TCC CTT CCA GGA CCA TAC TGC TAA-3'; wild type reverse, 5'-ACC CAA ACA GGC CAG TAC TCT A-3'; mutant forward, 5'-CTG CAG CCA AGC TAT CGA ATT CCT-3'; mutant reverse, 5'-TGC ATC ACC ACA CTT GGC GTA CTA-3'. The detection of a single PCR product of 230 bp confirmed the *IL30* gene knockout.

To demonstrate the *IFNγ* gene knockout, we used the following primers, provided by the Jackson Laboratories (Bar Harbor, ME, USA): wild type forward, 5'-AGA AGT AAG TGG AAG GGC CCA GAA G-3'; mutant forward, 5'-CCT TCT ATC GCC TTC TTG ACG-3'; common, 5'-AGG GAA ACT GGG AGA GGA GAA ATA T-3'. The detection of a single PCR product of 500 bp confirmed the *IFNγ* gene knockout.

Groups of 45 mice were subcutaneously injected with $2X10^5$ E0771 or AT-3 cells. For in vivo IFNγ blockade, mice were given 200 μg of neutralizing anti-IFNγ antibody (ATCC Cat# HB-170, RRID:CVCL_9233) two days before tumor cell injection[5] and every 4 day thereafter, until the end of the experiment. For histopathological, molecular and flow cytometry analyses, 15 mice from each group were euthanized when tumor sizes started to diverge (day 14, for E0771 tumors, and day 28, for AT-3 tumors), when it is more likely to identify differences in the tumor immune microenvironment.[6,7] The remaining 30 mice per group were kept until tumors reached 2000 mm^3, or evidence of suffering was observed, to evaluate tumor growth and the number of metastases.

Sample size and power calculation: with an overall sample size of 30 mice per group, a one-sided log rank test detected a difference of 30% in tumor growth or number of metastases between two groups, with a 90% power, at a 0.05 significance level (G*Power, RRID:SCR_013726).[8]

Randomization: randomization is not relevant to this study.

PCR array

The RNA was extracted by using the RNeasy Mini Kit (#74104, Qiagen, Hilden, Germany), and reverse-transcribed with the RT^2 First Strand Kit (#33040, Qiagen, Hilden, Germany), from cancer cells untreated or treated with recombinant rmIL30 (#7430-ML-010, R&D Systems, Minneapolis, MN, USA). The Real-Time PCR was run on a Qiagen Rotor Gene Q (Qiagen Rotor-Gene Q, RRID:SCR_018976), using the RT^2 Profiler Mouse Cancer Inflammation & Immunity Crosstalk PCR Array (#PAMM-181Z, Qiagen, Hilden, Germany) and RT^2 SYBR Green Master mix (#330501, Qiagen, Hilden, Germany). The results from each plate were normalized to the median value of a set of housekeeping genes. Changes in the gene expression were calculated using the ΔΔCt method. Results from experiments

performed in triplicate were pooled and analyzed with the manufacturer's software. A significant threshold of a 2-fold change in gene expression corresponded to a $p<0.001$.

Histopathology

Histology and single e double immunohistochemistry were performed as we reported,[4] by using the antibodies (Abs) listed in Supplementary Table S1.

The proliferation index, IFNγ expression, microvessel and immune cell counts were assessed by light microscopy at ×400 in an 85431.59 μm^2 field, on single immunostained sections, using Qwin image analysis software, version 2.7 (Leica QWin, RRID:SCR_018940). Six to eight high-power fields were analyzed for each section and three sections per sample were evaluated. Results are expressed as mean ± SD of positive cells per field (macrophages, Mac: F4/80$^+$; MDC: CD11b$^+$Gr-1$^+$; polymorphonuclear peutrophils, PMN: Ly-6G$^+$; T regulatory cells, Treg: Foxp3$^+$CD4$^+$; CD4$^+$T cells: CD3$^+$CD4$^+$; CD8$^+$T cells: CD3$^+$CD8$^+$; NK cells: NKp46$^+$), mean percentage ± SD of positive cells/number of total cells per field (to assess proliferation index by PCNA immunostaining), or mean percentage of positively stained areas/total area of the examined field (to assess IFNγ expression). Microvessels were identified as small tubes or circles marked by CD31 Ab and results were expressed as mean ± SD of positive vessels/field. Each slide was analyzed by two independent investigators, in a blind fashion, and there was an almost perfect agreement (kappa value = 0.82) between their evaluations.

Confocal microscopy

Immunofluorescent stainings were performed on frozen sections of tumors developed from E0771 or AT-3 cells.

Slides were incubated with PE-conjugated Ab against CD126 (IL6Ra) (Thermo Fisher Scientific Cat# 12-1261-80, RRID:AB_996658) or with APC-conjugated Ab anti-CD130 (gp130) (Thermo Fisher Scientific Cat# 17-1302-82, RRID:AB_10670874), for 30 min. at room temperature. Subsequently, nuclei were counterstained with DAPI Solution (#62248, Thermo Fisher Scientific, Waltham, MA, USA) for 4 minutes and slides were analyzed under an LSM 510 Meta confocal microscope (Zeiss, Oberkochen, Germany; RRID:SCR_018062).

Flow cytometry

After dissociation in a single cell suspension, E0771 and AT-3 cells were fixed with 1% paraformaldehyde and incubated for 30 minutes with anti-mouse CD126 (IL6Ra) PE (Thermo Fisher Scientific Cat# 12-1261-80, RRID:AB_996658) and anti-mouse CD130 (gp130) APC (Thermo Fisher Scientific Cat# 17-1302-82, RRID:AB_10670874) Abs.

Tumor and spleen samples (five per group) were cut into small pieces and crushed through a Corning cell strainer (size 40 µm). The resulting cells were resuspended in PBS and stained with 7-AAD prior to surface staining with the following anti-mouse Abs: anti-CD3 BD Horizon BV 421 (Cat# 564008, RRID:AB_2732058), anti-CD4 APC (Cat# 553051, RRID:AB_398528), anti-CD8a APC-H7 (Cat# 560182, RRID:AB_1645237), anti-CD25 FITC (Cat# 553071, RRID:AB_394603), anti-NKp46 PerCP-Cy™5.5 (Cat# 560800, RRID:AB_2034018) (all from BD Biosciences), anti-CD11b PE (Cat# 101208, RRID:AB_312791) and anti-Gr-1 APC (Cat# 108412, RRID:AB_313377) (both from BioLegend, San Diego, CA, USA). Cells were then washed, fixed and permeabilized with Flow Cytometry Fixation/Permeabilization Buffer I (R&D Systems Cat# FC007, RRID:AB_10386921), prior to staining with anti-Foxp3 PE (Cat# 560408, RRID:AB_1645251) and anti-IFNγ APC-Cy™7 (Cat# 561479, RRID:AB_10898181) (both from BD Biosciences). For the analysis of IFNγ production by tumor infiltrating cells, we

applied the following gating strategy: lymphocytes were identified on an FSC-A/SSC-A dot-plot and then analyzed for their CD3 expression. Then, $CD4^+$ and $CD8^+$compartments were identified within the $CD3^+$ gate and INFγ expression was measured (in terms of percentage and expression) in both compartments. Subsequently, the compartment of $NKp46^+$cells was gated within the $CD3^-$cell subset and the INFγ expression was evaluated, in terms of percentage and expression.

Acquisition was performed using a BD Scientific Canto II Flow Cytometer (RRID:SCR_018056) and data were analyzed using FlowJo software (FlowJo, RRID:SCR_008520). Isotype controls were used to assess the background and experiments were performed in triplicate.

Statistics

For *in vitro* and *in vivo* studies, between-group differences were assessed by Student's *t*-test, Fisher's exact test or ANOVA, followed by Tukey HSD test.

Survival curves were constructed using the Kaplan-Meier method and survival differences were analyzed by the log-rank test. All statistical tests were evaluated at an α level of 0.05, using Stata version 13 (StataCorp, College Station, TX, USA; RRID:SCR_012763).

REFERENCES

1. Stewart TJ, Abrams SI. Altered immune function during long-term host-tumor interactions can be modulated to retard autochthonous neoplastic growth. *J Immunol.* 2007;179:2851-2859.
2. Johnstone CN, Smith YE, Cao Y, et al. Functional and molecular characterisation of EO771.LMB tumours, a new C57BL/6-mouse-derived model of spontaneously metastatic mammary cancer. *Dis Model Mech.* 2015;8:237-251.

3. Zhang S, Liang R, Luo W, et al. High susceptibility to liver injury in IL-27 p28 conditional knockout mice involves intrinsic interferon-γ dysregulation of CD4+ T cells. *Hepatology.* 2013;57:1620-1631.
4. Dalton DK, Pitts-Meek S, Keshav S, Figari IS, Bradley A, Stewart TA. Multiple defects of immune cell function in mice with disrupted interferon-gamma genes. *Science.* 1993;259:1739-1742.
5. Murillo O, Arina A, Hervas-Stubbs S, et al. Therapeutic antitumor efficacy of anti-CD137 agonistic monoclonal antibody in mouse models of myeloma. *Clin Cancer Res.* 2008;14:6895-6906.
6. Oh J, Magnuson A, Benoist C, Pittet MJ, Weissleder R. Age-related tumor growth in mice is related to integrin α 4 in CD8+ T cells. *JCI Insight.* 2018;3:e122961.
7. Davidson S, Efremova M, Riedel A, et al. Single-Cell RNA Sequencing Reveals a Dynamic Stromal Niche That Supports Tumor Growth. *Cell Rep.* 2020;31:107628.
8. Faul F, Erdfelder E, Buchner A, Lang AG. Statistical power analyses using G*Power 3.1: tests for correlation and regression analyses. *Behav Res Methods.* 2009;41:1149-1160.
